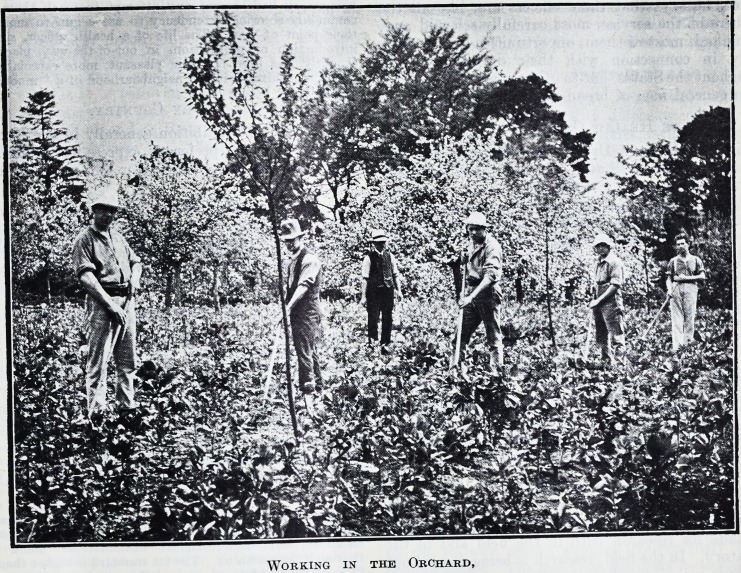# Seamen and Tuberculosis

**Published:** 1924-10

**Authors:** 


### SEAMEN AND TUBERCULOSIS.

### KING GEORGE'S SANATORIUM FOR SAILORS.

In our July issue, in connection with the Duke of York's visit to the branch of the Seamen's Hospital Society at the Tilbury Docks, we printed some details of the manifold activities of this vigorous Corporation on behalf of the sick and injured seamen. We are now able to give an account of the sanatorium for seamen suffering from tuberculosis, which is under the care of the same Society at Bramshott.

### The Need of the Sailor.

For many years before 1921, when the sanatorium was opened by the Duke of York, an urgent need had existed for affording succour to mariners afflicted with consumption. Tuberculosis is not only widely prevalent, but is actually on the increase among seamen. Apart from the fact that there are not sufficient sanatoria beds available for the number of patients suffering from tuberculosis, the mariner often finds the greatest difficulty in gaining admission to any of the existing sanatoria, owing, in a great measure, to the fact that he can frequently show no domicile. The late Lord Beresford, himself a great sailor, once said that: "A sailor's life is the hardest to which the destiny of man can wed him." And, strange though it may seem, considering the open-air life the sailor leads, he is frequently a victim of tuberculosis. This matter caused considerable anxiety to the Seamen's Hospital Society, for, although an open-air ward was maintained on the roof of the old *Dreadnought* at Greenwich, it did not prove as efficient as the medical staff would have wished, and it became apparent that to combat consumption a sanatorium was an absolute necessity. Lord Inchcape, therefore, with the public spirit which characterises even all his business ventures, raised the large sum of £100,000 to establish, equip and endow an up-to-date sanatorium, and the King commanded that it should bear his Royal name.

### The Site and the Building.

Bramshott Place is situated on the south side of the Portsmouth Road, four miles south of Hindhead and about forty miles from London. Upon a light and easily-worked soil, with gravel beneath, the sanatorium is built at the top of a wooded slope facing south. The old house, the foundations of which date as far back as the sixteenth century, has been admirably adapted as quarters for the nursing staff. The plans of the architect, Mr. S. Hamp, A.R.I.B.A., provided for the accommodation of the patients in a two-storey pavilion of fireproof reinforced concrete faced with stucco and roofed with tiles of a charming mellow red hue. The centre of the building consists of single and double cubicles, while at the sides are two wards each containing ten beds. The cubicles have iron-framed double-casement doors, with overhead and side lights, the whole front of the cubicle being arranged to open. A duty room and ward kitchen are provided on each floor, with glass bays from which the whole length of the floor can be observed. On each floor is a wide verandah, the upper one being only partially covered to permit of intensive sun treatment. The lower floor, in addition to the verandah in front, has a service corridor behind, with doors to each cubicle, which is most useful for the conveyance of food and stores and for various nursing details.

The Chicken Farm.

RECREATION.

This system of cubicles is found to have very definite advantages in this form of institution, for not only are advanced cases less disturbed, and thereby obtain more rest and quiet, but it is found that early cases are more easily controlled under this system. The building is well equipped with a medical superintendent's office, operating theatre, laboratory, X-ray department, plaster room, sputum room, store rooms, etc. Throughout the interior is laid a composition flooring of an attractive red shade, while the walls are enamelled cream. A hot-water radiator system is installed for the warming of certain cubicles, and the large wards have open brick fireplaces where cheerful log fires are lighted in wet weather. Two recreation rooms and an excellent library —most essential adjunct to a sanatorium of this kind—are part of the equipment of the house. The grounds of the estate are seventy acres in extent and are very beautiful. In addition to lawns and flower gardens there are orchards, kitchen gardens, and a chicken farm. Men who are on the road to recovery are instructed in various branches of light agricultural work, according to their capacity.

WORKING IN THE ORCHARD.

THE STAFF.

Owing to the large number of cases confined to bed, both advanced pulmonary and less advanced non-pulmonary cases for whom prolonged recumbency is necessary, the nursing staff is somewhat larger than in the ordinary sanatorium of similar size. Besides the matron there are two sisters, four trained staff nurses and eight probationers. Of these one staff nurse and one probationer are on night duty. The domestic staff, including ward-maids and laundry hands (the sanatorium has its own laundry) numbers twelve. The male staff consists of two engineers, three gardeners, two porters and a scullery boy. The medical superintendent is Dr. J. E. Wood, late of the Northamptonshire Sanatorium, who works with the consulting staff, which consists of Sir Henry Gauvain, Dr. Charles Sundell and Mr. Gilbert Smith. The matron is Miss Williams, R.R.C., and the secretary for the Society is Paymaster Captain F. Gerty, C.M.G., R.N. In 1923, 131 patients were admitted. The average number of days that each patient was in residence was 175, and the average cost per patient per week was £2 14s. 8d. It will be seen, therefore, that King George's Sanatorium for Sailors fulfils with some success the purpose for which it was founded. Here in the pure upland air, far from their restless life on the ocean, phthisical sailors are tended, having the fresh country food and produce which do so much to bring about their recovery. Not only does the Institution treat those who may recover and return to their calling, but it brings comfort and relief to those in the more advanced stages of the disease who may never be restored to health. Our indebtedness to the sailor is great indeed. Our very existence depended on him during the war, and he is apt sometimes to pass out of our remembrance, so silently and out of sight does he work.

## Figures and Tables

**Figure f1:**
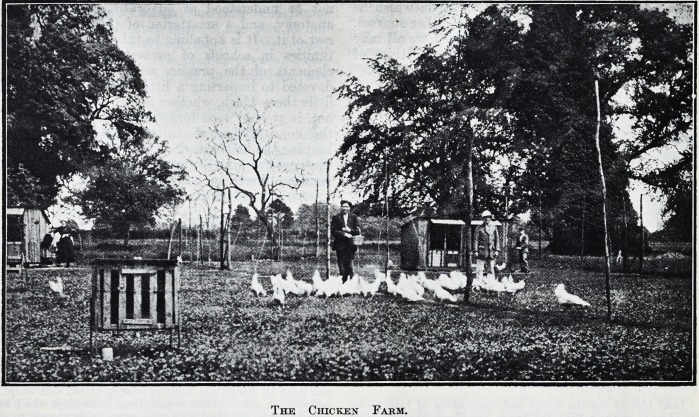


**Figure f2:**